# A flexible arched artificial photoreceptor constructed by photodeformable liquid crystal polymers and its application in vision restoration

**DOI:** 10.1002/smo2.70030

**Published:** 2026-01-19

**Authors:** Yumeng Jiang, Bo Peng, Jinyu Ma, Feng Pan, Jia Wei, Lang Qin, Cheng Sun, Yanlei Yu

**Affiliations:** ^1^ State Key Laboratory of Molecular Engineering of Polymers, College of Smart Materials and Future Energy Fudan University Shanghai China; ^2^ Key Laboratory of Neuroregeneration of Jiangsu and Ministry of Education, Co‐Innovation Center of Neuroregeneration, NMPA Key Laboratory of Research and Evaluation of Tissue Engineering Technology Products, School of Medicine Nantong University Nantong China

**Keywords:** artificial photoreceptors, liquid crystal polymers, piezoelectric materials, vision restoration

## Abstract

Artificial photoreceptors capable of eliciting neural responses offer a promising strategy for restoring vision in individuals with retinal degenerative diseases. However, stimulating neurons under low‐intensity light remains a critical challenge, which significantly hampers their practical application. Here, a flexible arched artificial photoreceptor with strong photoelectric response under weak light is constructed by photodeformable liquid crystal polymers (LCPs) and polyvinylidene fluoride‐trifluoroethylene (P(VDF‐TrFE)). The light‐stress‐electricity conversion arising from photo‐induced stress of LCPs and the piezoelectric effect of P(VDF‐TrFE) is significantly enhanced by the arched structure, which induces stress concentration. Hence, the open‐circuit voltage reaches up to 17.51 ± 0.60 V under 8 mW cm^−2^ light irradiation, which is 21 times higher than that of the planar structure (0.79 V), with a 10‐fold reduction in light intensity. By analyzing the voltage of units, the pixelated matrix of artificial photoreceptors is capable of imitating complex visual functions including light detection, pattern recognition and information decoding. Notably, the flexibility and biocompatibility endow this artificial photoreceptor with great potential in artificial retinal applications. Blind rats implanted with this artificial photoreceptor are demonstrated to exhibit restored visual responses. This study presents a novel approach to fabricating artificial photoreceptors which are sensitive to weak light and provides new insights for the applications of LCPs in implantable devices.

## Graphical abstract

A flexible arched artificial photoreceptor with high stability and biocompatibility was fabricated by integrating liquid crystal polymers with polyvinylidene fluoride‐trifluoroethylene. This device exhibits robust photoelectric responses under low‐intensity illumination and demonstrates efficacy in restoring visual responses in blind rats following implantation.

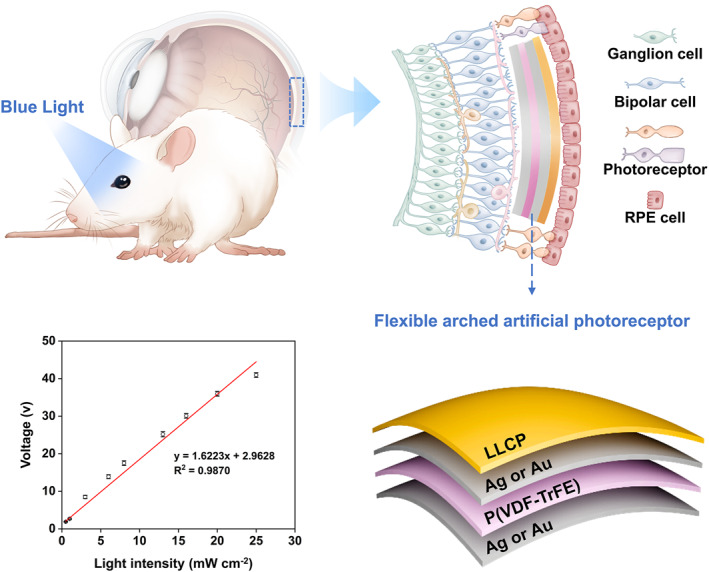

## 1 INTRODUCTION

Photoreceptors (i.e., rod cells and cone cells) are vital in visual transmission, which convert optical signals into neural electrical stimuli via photopigments.^[1]^ Individuals with retinal degenerative diseases such as retinitis pigmentosa and age‐related macular degeneration suffer from severe visual impairments or blindness due to damage or loss of photoreceptors,^[2]^ while the inner retinal neurons largely remain intact.^[3]^ Bioengineering visual systems using subretinal prosthesis such as the microelectrode array[^4^, ^5^, ^6^] and photodiode electrode array[^7^, ^8^] have been demonstrated to effectively stimulate neurons (primarily bipolar cells) and elicit visual responses. However, these systems require cumbersome wiring and involve complex surgical procedures. Therefore, recent research has focused on intelligent materials that are capable of photoelectric conversion[^2^, ^9^, ^10^, ^11^, ^12^, ^13^, ^14^, ^15^, ^16^, ^17^, ^18^, ^19^, ^20^] to construct artificial photoreceptors as promising substitutes for lost photoreceptors. Nevertheless, inorganic photoelectric materials face challenges of mechanical incompatibility with soft neural tissues, while their organic counterparts pose potential risks of biological toxicity arising from photoelectrochemical reactions involving photoexcited electrons. Besides, photothermal‐pyroelectric composites exhibit robust and strong photoelectric response but may cause thermal damage to tissues as implants.

A novel type of composite based on light‐stress‐electricity conversion and composed of photodeformable materials and piezoelectric materials provides a feasible strategy to address aforementioned limitations.[^21^, ^22^, ^23^] Azobenzene‐containing liquid crystal polymers (LCPs) generate significant photo‐induced stress by amplifying microscopic geometric changes of azobenzene moieties into macroscopic deformation on the basis of the cooperative effect of the mesogens,[^24^, ^25^, ^26^, ^27^, ^28^] thus standing out among photodeformable materials.[^29^, ^30^, ^31^] The open‐circuit voltage of a neuron‐readable artificial photoreceptor composed of LCPs and polyvinylidene fluoride‐trifluoroethylene (P(VDF‐TrFE)) reaches up to 0.79 ± 0.02 V,^[31]^ which is 19 times higher than the maximum voltage reported in other works based on light‐stress‐electricity conversion.^[22]^ However, a strong light intensity is needed for excitation, which may restrict the practical application of artificial photoreceptors under weak light. The performance of a device is typically determined by both the material properties and structural design. LCPs and P(VDF‐TrFE), with intrinsic flexibility, allow for three‐dimensional structural configurations, which hold great potential to further enhance the photoelectric response in weak‐light conditions.

Designing special geometric structures of piezoelectric polymers has shown excellent performance in improving the piezoelectric response.[^32^, ^33^, ^34^, ^35^, ^36^, ^37^] Dong and co‐workers fabricated a wave‐shaped P(VDF‐TrFE)/FeSiB composite with a better piezoelectric effect compared with that of the composite in a planar structure. The special wavy shape enhances the electromechanical coupling, thus increasing the output voltage by 1.5–3 times.^[32]^ Chu and co‐workers increased the effective piezoelectric response of P(VDF‐TrFE) arched film by 70‐fold compared with that of the planar film under the same pressure, mainly because of the stress concentration at the outer rim of the arch film.^[37]^ In planar structures, the effective piezoelectric response is primarily governed by the longitudinal piezoelectric response (d_33_ mode), while in three‐dimensional structures, large transverse piezoelectric responses (d_31_ mode and d_32_ mode) are activated owing to the stress concentration effect, greatly increasing the voltage output by devices.^[37]^ Therefore, constructing three‐dimensional structures of devices is a promising strategy for enhancing the photoelectric conversion of artificial photoreceptors, enabling responsiveness under the illumination of weak light.

**FIGURE 1:**
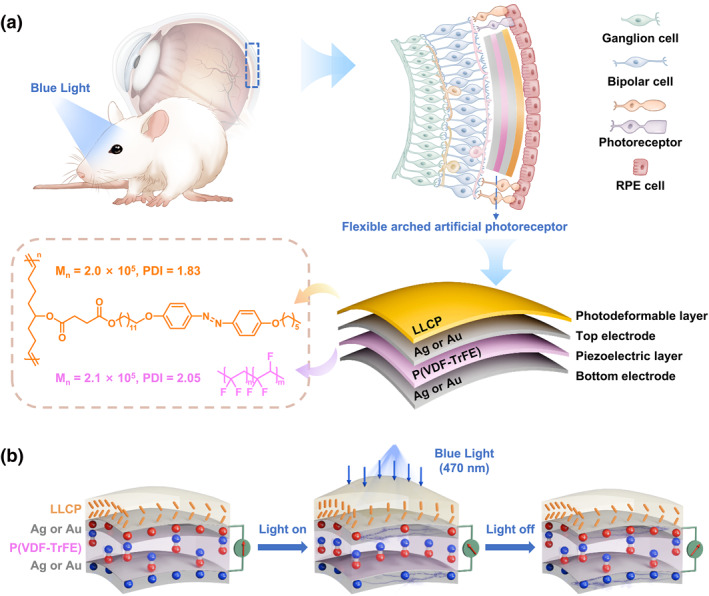
A flexible arched artificial photoreceptor that converts light into electric signals and restores visual responses of blind rats. (a) Schematic illustration to show the structure and implantation of the flexible arched artificial photoreceptor. (b) Schematic illustration to show the light‐stress‐electricity conversion of the flexible arched artificial photoreceptor.

Herein, we report a flexible arched artificial photoreceptor composed of the photodeformable LCP and piezoelectric material P(VDF‐TrFE), which outputs large electric signals under weak light based on light‐stress‐electricity conversion. The photoelectric response is improved by a unique design of the arched structure, which leads to stress concentration. Consequently, the photo‐induced stress, which is concentrated at the outer rim of the LCP film, is transmitted to the P(VDF‐TrFE) film and converted to significant electric signals. The open‐circuit voltage reaches up to 17.51 ± 0.60 V under 470 nm light at 8 mW cm^−2^. Compared with previously reported work using light‐stress‐electricity conversion, the open‐circuit voltage increases 21 times with a 10‐fold reduction in light intensity.^[31]^ The pixelated matrix which integrates artificial photoreceptors is able to imitate photoreceptors in the natural retina to replicate complex visual functions. Moreover, we evaluated the biocompatibility and investigated the potential of flexible arched artificial photoreceptors for subretinal implantation in blind rats, aiming to replace the dysfunctional photoreceptor layer comprising rod and cone cells (Figure 1a). Both electrophysiological measurements and behavioral analyses revealed that the implanted artificial photoreceptors restored the visual responses of blind rats.

## 2 RESULTS AND DISCUSSION

### 2.1 Composite fabrication and structure optimization

In order to fabricate a flexible and biocompatible artificial photoreceptor that can be implanted, we constructed a four‐layer structure, including a photodeformable layer, a piezoelectric layer, and two conductive layers (Figure 1a). The top layer is composed of a linear liquid crystal polymer (LLCP) film that converts light into stress. The LLCP, which contains flexible backbones and azobenzene moieties, self‐assembles into a highly ordered structure due to the molecular cooperation effect of liquid crystal and free volume provided by long spacers (Figure 1a), and undergoes a fast and reversible deformation under light illumination.^[38]^ Moreover, the LLCP is easily processed into 3D structures or combined with other layers by heating above the clearing point owing to the linear structure. P(VDF‐TrFE) is selected as the piezoelectric layer and sandwiched between two layers of Ag electrodes, which exhibit great conductivity to promote charge accumulation. In the implantation experiment, the Ag electrodes were replaced with Au electrodes to prevent the impact of the in vivo chloride ion environment. Flexible arched artificial photoreceptors generate large photo‐induced electric signals due to the light‐stress‐electricity conversion. Under the irradiation of 470 nm light, the azobenzene moieties of LLCP undergo oscillation of *trans‐cis‐trans* isomerization, which causes volume expansion and generates large photo‐induced stress. Because the absorption bands of the *n*‐π* transition of the *trans* form and the *n*‐π* transition of the *cis* form overlap around 470 nm, as shown in the UV‐vis absorption spectrum of LLCP (Supporting Information S1: Figure S1). After the stress is transferred to the piezoelectric layer, the change of dipole density in P(VDF‐TrFE) layer leads to the redistribution of charges in the electrodes; therefore, electric signals could be detected (Figure 1b).^[12]^ When the light is off, the dipole density in P(VDF‐TrFE) returns to its initial state again.

**FIGURE 2:**
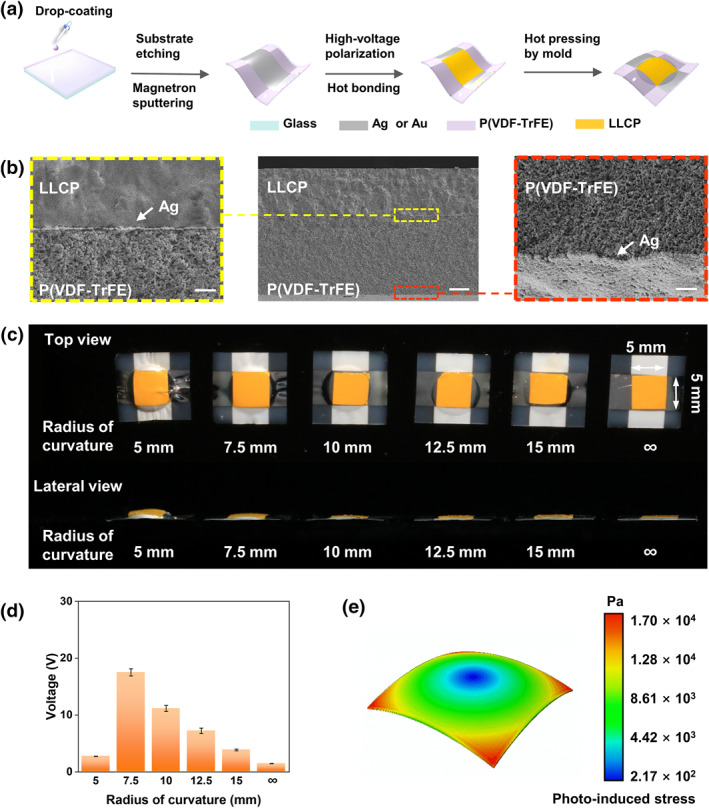
The impact of structure on the electrical signals output by flexible arched artificial photoreceptors. (a) Schematic illustration to show the fabrication of the flexible arched artificial photoreceptor. (b) The SEM photograph of the cross‐section of the flexible arched artificial photoreceptor. Scale bar: 1, 1, and 10 μm. (c) Photographs of the flexible arched artificial photoreceptors with different radii of curvature. (d) Plot showing the open‐circuit voltage of flexible arched artificial photoreceptors with different radii of the curvature under 470 nm light irradiation (8 mW cm^−2^). (e) Stress distribution of the flexible arched artificial photoreceptor with the radius of curvature at 7.5 mm under 470 nm light irradiation (8 mW cm^−2^) calculated by finite element analysis.

Stability of the structure is essential for ensuring reliable and high‐performance operation of functional devices. During the fabrication of the flexible arched artificial photoreceptor, the electrode was deposited on both sides of the P(VDF‐TrFE) layer by magnetron sputtering to enhance the charge accumulation. After high‐voltage polarization, the LLCP layer was bonded on the Ag electrode layer by heating and the composite was pressed in a mold to form the arched structure (Figure 2a). The cross‐sectional morphology of the artificial photoreceptor shown in the SEM photograph reveals that the four‐layer structure is tightly bonded with no obvious cracks at the junction between layers, which reduces the stress loss during transmission (Figure 2b). We prepared a series of arched artificial photoreceptors with different radii of curvature and investigated their forming stability (Figure 2c). The shape of the arch structure 1 month after hot pressing was measured by ultra‐depth 3D microscopy to determine whether the radius of curvature was consistent with the design (Supporting Information S1: Figure S2). Results show that when the radius of curvature is greater than or equal to 7.5 mm, the measured heights and theoretical heights are essentially the same, indicating that the shape of the arched artificial photoreceptor prepared by hot pressing with arched mold is precisely controlled and stably maintained. However, obvious wrinkles and partial overlap on artificial photoreceptors with a radius of curvature of 5 mm indicated that the integrity of the arch structure with a small radius of curvature was damaged.

The radius of curvature is of great importance to piezoelectric devices, so we further investigated the impact of radius of curvature on the electrical signals output by flexible arched artificial photoreceptors under light irradiation (470 nm, 8 mW cm^−2^). The open‐circuit voltage gradually increases with decreasing radius of curvature and reaches a peak value of 17.51 V at a radius of curvature of 7.5 mm (Figure 2d). This trend is attributed to the elevated stress concentration that amplifies the non‐uniform distribution of photo‐induced stress generated by LLCP and boosts the effective piezoelectric response. However, when the radius of curvature is reduced to 5 mm, the flexible arched artificial photoreceptor exhibits a markedly decreased open‐circuit voltage (2.74 V), primarily due to the compromised integrity of the arched structure. The finite element analysis was performed to simulate the stress distribution of the flexible arched artificial photoreceptor with the radius of curvature at 7.5 mm under 470 nm light irradiation (8 mW cm^−2^), revealing that the photo‐induced stress is concentrated at the outer rim of the artificial photoreceptor (Figure 2e). This study indicates that the arched structure is essential for enhancing the electrical output of artificial photoreceptors.

### 2.2 Photoelectric response of the flexible arched artificial photoreceptor

**FIGURE 3:**
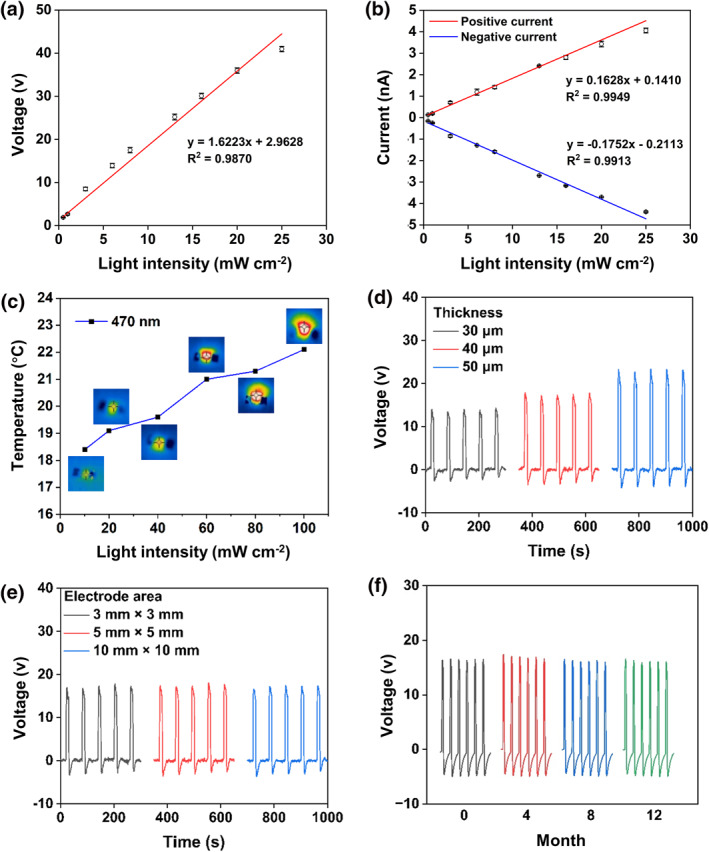
Photoelectric performances of flexible arched artificial photoreceptors (radii of curvature: 7.5 mm). (a) The open‐circuit voltage and (b) the short‐circuit current of flexible arched artificial photoreceptors under 470 nm light at different intensities. (c) Plot showing the temperature of the flexible arched artificial photoreceptor with different light intensities. The inset is the infrared thermography with different light intensities. (d) The open‐circuit voltage of the flexible arched artificial photoreceptor with different thickness of the piezoelectric layer. The radius of curvature is 7.5 mm. The size of the electrodes is 5 × 5 mm. The light intensity is 8 mW cm^−2^. (e) The open‐circuit voltage of the flexible arched artificial photoreceptor with different sizes of the electrodes. The radius of curvature is 7.5 mm. The thickness of the piezoelectric layer is 40 μm. The light intensity is 8 mW cm^−2^. (f) Stability test of the flexible arched artificial photoreceptor in 12 months. The light intensity is 8 mW cm^−2^.

It is feasible to regulate the electrical output of flexible arched artificial photoreceptors by controlling the light intensity, owing to the linear relationship between light intensity and photo‐induced stress which has been studied in our previous work. The photo‐induced stress of the planar LLCP film increases linearly with increasing intensity of 470 nm light and reaches a plateau (0.3 MPa) at 80 mW cm^−2^. The saturation value is comparable to the stress generated by vertebrate muscle fibers (0.35 MPa).^[31]^ When the light intensity increases from 0.5 to 25 mW cm^−2^, both the peak value of the open‐circuit voltage and short‐circuit current increase and show a good linear relationship with light intensity (Figure 3a,b), which is beneficial for identifying light signals with different intensities. Notably, flexible arched artificial photoreceptors exhibit excellent photoelectric conversion capability. The open‐circuit voltage reaches up to 40.96 ± 0.61 V under 25 mW cm^−2^ light (Figure 3a), which is approximately equal to the maximum voltage (42.4 V at 100 mW cm^−2^) that has been reported in the case of polymer‐based composites used in subretinal prostheses,^[9]^ with a 4‐fold decrease in light intensity. Besides, the open‐circuit voltage reaches up to 17.51 ± 0.60 V under 8 mW cm^−2^ light (Figure 3a), which is 21 times higher than the maximum voltage that has been reported in the photo‐stress‐electricity conversion system (0.79 V at 80 mW cm^−2^),^[31]^ while reducing the light intensity to only 1/10. Even under 0.5 mW cm^−2^ weak light, the open‐circuit voltage reached up to 1.90 ± 0.09 V. The outstanding photoelectric conversion performance allows the artificial photoreceptor to overcome the limitation of requiring strong light illumination, creatively expanding the application scenarios and enabling its use in the daily environment.

To exclude the influence of the photothermal effect, we exposed the flexible arched artificial photoreceptor to 470 nm light and recorded temperature changes using an infrared thermal imager. When the light intensity increases to 100 mW cm^−2^, the temperature only rises by 4.1°C, which is not sufficient to induce the phase transition and deformation of LLCP by the photothermal effect (Figure 3c). The thickness of the piezoelectric layer affects the number of oriented dipoles; thus, we prepared P(VDF‐TrFE) films with varying thicknesses to investigate the influence on the open‐circuit voltage. Under 470 nm blue light illumination at an intensity of 8 mW cm^−2^, the open‐circuit voltage of the flexible arched artificial photoreceptor increases with the thickness of the piezoelectric layer (Figure 3d). Dipoles near the film surface are constrained by surface tension, making it difficult for them to reorient during polarization. Increasing the thickness improves the proportion of effectively reoriented dipoles, thereby enhancing polarization strength. However, when the thickness exceeds 40 μm, the P(VDF‐TrFE) film becomes relatively stiff, which may complicate subretinal implantation and potentially cause bleeding. Therefore, the thickness of 40 μm is considered optimal for the P(VDF‐TrFE) film as a part of the flexible arched artificial photoreceptor. Besides, to achieve structural compatibility with natural retina, we need to minimize the device thickness as much as possible, so we selected 20‐μm‐thick LLCP films that combine favorable mechanical properties with substantial photo‐induced stress to construct the device. In addition, we explored the influence of electrode size on the open‐circuit voltage of the flexible arched artificial photoreceptor. The results indicate that the open‐circuit voltage remains unaffected by variations in electrode size as the charge density on the top and bottom surfaces of the piezoelectric layer is independent of it (Figure 3e). This finding is advantageous for the miniaturization and integration of electrodes enabling the fabrication of array‐type devices. The stability and repeatability of signals are crucial for implantable devices. The fatigue resistance test of flexible arched artificial photoreceptors shows that the open‐circuit voltage remains around 17.5 V over 500 cycles of light illumination (470 nm, 8 mW cm^−2^), proving the repeatability and stability of the electric signal output (Supporting Information S1: Figure S3). Besides, the results of open‐circuit voltage tests conducted every 3 months reveal that the open‐circuit voltage remains stable even after 12 months, providing assurance for the long‐term service life of flexible arched artificial photoreceptors (Figure 3f).

### 2.3 Imitation of sophisticated visual functions

A single flexible arched artificial photoreceptor detects only light on‐ or off‐state. However, a pixelated matrix of such artificial photoreceptors, integrated to emulate human retinal photoreceptors, achieves more complex visual functions. We fabricated 3 × 3 pixelated matrix and 10 × 10 pixelated matrix by utilizing electrode photomasks for electrode deposition and arched molds for hot pressing (Supporting Information S1: Figures S4 and S5). The schematic illustration of the pixelated matrix is shown in Supporting Information S1: Figures S6 and S7. To prevent interference between pixel units, we designed non‐overlapping electrodes (Supporting Information S1: Figure S8). The feasibility of the matrix was validated by testing crosstalk in a 3 × 3 pixelated matrix (Supporting Information S1: Figure S9a). The open‐circuit voltage of the central illuminated pixel reaches the volt (V) range (red), while that of adjacent unilluminated pixels remains in the millivolt range, exhibiting a difference of three orders of magnitude. The difference remains almost constant after an increase in light intensity (Supporting Information S1: Figure S9b), which demonstrates that different pixels do not interfere with one another in the pixelated matrix, ensuring the accuracy of functions.

**FIGURE 4:**
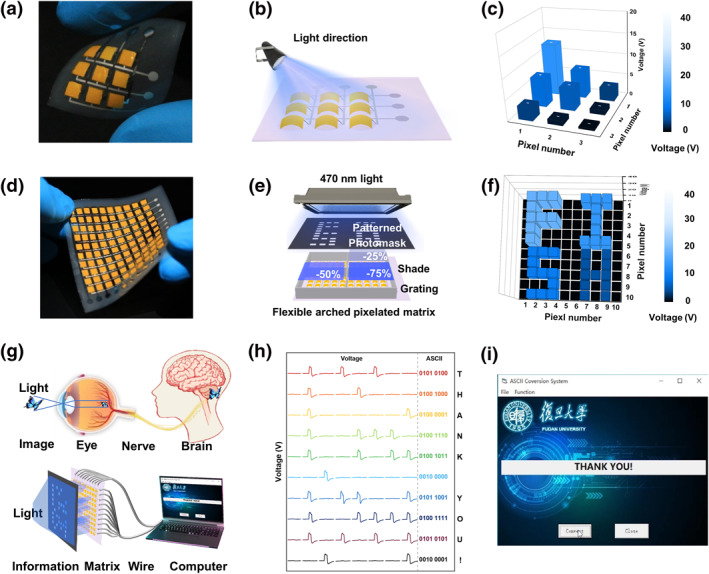
Complicated visual functions achieved by the flexible arched pixelated matrix. (a) Photograph of the 3 × 3 flexible arched pixelated matrix. (b) Schematic illustration to show the light direction of the 3 × 3 flexible arched pixelated matrix. (c) The open‐circuit voltage of the 3 × 3 flexible arched pixelated matrix to identify the light direction. (d) Photograph of the 10 × 10 flexible arched pixelated matrix. (e) Schematic illustration of the complex patterns and brightness recognition. (f) The open‐circuit voltage of the 10 × 10 flexible arched pixelated matrix to identify complex patterns and brightness. (g) Schematic illustration of the human's vision process and simulation of the human's vision by the flexible arched pixelated matrix. (h) The open‐circuit voltage of the flexible arched pixelated matrix and (i) the corresponding ASCII conversion results.

Complex functions can be achieved by analyzing the voltage of units on the pixelated matrix. The proximity of pixels to the light source directly influences their open‐circuit voltages, with closer pixels exhibiting higher voltages, thus creating a voltage gradient across the entire matrix. For 3 × 3 flexible arched pixelated matrix (Figure 4a), the direction of the light source is identified based on the value and distribution of the open‐circuit voltage (Figure 4b,c and Supporting Information S1: Figure S10). By establishing a relationship between the open‐circuit voltage of pixel points and light intensity, the 10 × 10 flexible arched pixelated matrix (Figure 4d) is expected to identify complex patterns with different brightness. As shown in Figure 4e, the matrix is divided into four regions with different light intensities using gratings and shades with different blue light transmittances. Open‐circuit voltages of the four regions are plotted on the bar chart (Figure 4f), allowing for the clear identification of the “FISH” pattern with gradations in brightness.

To emulate the human vision process, we utilized a patterned mask to impart specific light information and employed an artificial photoreceptor pixelated matrix as a retinal substitute for light‐electric signals conversion. The electric signals were then transmitted to a computer via wires for decoding the light‐carrying information (Figure 4g). The high and low voltages generated by the pixels were defined as “1” and “0,” respectively. Using ASCII, light signals carrying information are interpreted through an ASCII conversion program. Upon illuminating the pixelated matrix with a 470 nm light (8 mW cm^−2^) through a patterned photomask encoded with ASCII, the values of open‐circuit voltage in the illuminated and blocked areas are approximately 17.5 and 0.7 V, respectively. As shown in Figure 4h, the voltage sequence of the 8 pixels in the first row corresponds to “01010100” in ASCII, which is translated into the character “T.” Similarly, each sequence corresponds to a character. Upon inputting the binary file containing the voltages of 80 pixels into our ASCII conversion program, we successfully deciphered the message “THANK YOU!” (Figure 4i).

### 2.4 Biocompatibility validation and vision restoration assessment of blind rats

The outstanding light‐stress‐electricity conversion capabilities and flexibility of the arched artificial photoreceptor render it a promising candidate for implantable retinal prostheses. To preliminarily assess the biocompatibility of this artificial photoreceptor, cell‐based assays were conducted. To promote cell adhesion and growth on the material surfaces, the LLCP film, P(VDF‐TrFE) film and glass substrate were first subjected to hydrophilic surface modification (Supporting Information S1: Figure S11). Human umbilical vein endothelial cells (HUVECs) were then seeded onto the modified films as well as a glass substrate as a control group. The cell morphology was observed by confocal laser scanning microscopy (Supporting Information S1: Figure S12). On day 0, the cells exhibited a round morphology, indicating that they had not yet adhered to the substrate. On day 4, cells showed good adherence and increased number; furthermore, specific fluorescence detection of filamentous actin (F‐actin) was performed. F‐actin was stained red by rhodamine phalloidin to show the cytoskeleton, while cell nuclei were dyed blue by DAPI. Confocal microscopy images reveal normal cytoskeletal morphology and a high confluency (Supporting Information S1: Figure S13a). Semi‐quantitative analysis of fluorescence images from three groups using ImageJ showed no significant difference in F‐actin expression levels among cells on LLCP, P(VDF‐TrFE), and glass (Supporting Information S1: Figure S13b). The number of HUVECs counted by hemocytometer and CCK‐8 assay shows that HUVECs could proliferate normally on both LLCP and P(VDF‐TrFE) films, suggesting that materials have no adverse effect on cell viability (Supporting Information S1: Figure S14). To evaluate the stability of the materials under physiological conditions, LLCP and P(VDF‐TrFE) films were immersed in physiological saline (0.9% NaCl, pH 7.4) at 37°C for 1 month. The FTIR‐ATR spectra show that the chemical structures of both films remain unchanged after immersion with no detectable signs of degradation (Supporting Information S1: Figure S15). The results above suggest that LLCP and P(VDF‐TrFE) films exhibit favorable biocompatibility and maintain good stability in a simulated physiological environment.

**FIGURE 5:**
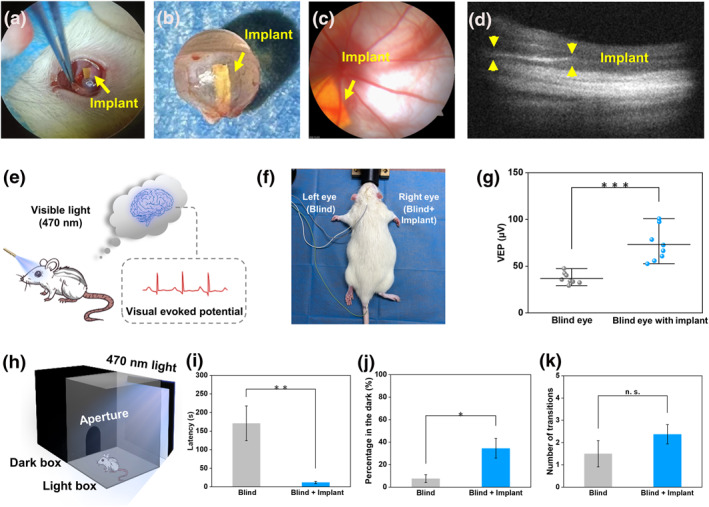
Implantation of artificial photoreceptors and light perception of blind rats with implants. (a) Implanting surgery of the flexible arched artificial photoreceptor. (b) Photograph of the rat eyeball implanted with the flexible arched artificial photoreceptor. (c) The fundus photograph of the rat eye with the flexible arched artificial photoreceptor implanted for 2 weeks. (d) The OCT image of the rat eye with the flexible arched artificial photoreceptor implanted for 2 weeks. (e) Schematic illustration that shows the electrophysiological assessment of the visual response of rats. (f) Photograph of the VEPs test. (g) VEPs in control group (36.89 ± 1.98 μV) and implanted group (73.15 ± 6.00 μV) under the illumination of 470 nm light (8 mW cm^−2^) (mean ± SEM, *n* = 8, paired *t* test, ***p < 0.001). (h) The light‐dark box test revealing the restoration of light sensitivity of blind rats with implants. (i) The latency of blind rats (170.88 ± 46.69 s) and blind rats with implants (12.00 ± 2.87 s) (mean ± SEM, *n* = 8, unpaired *t* test, **p < 0.01). (j) The percentage of time spent in the dark box of blind rats (7.58 ± 3.46%) and blind rats with implants (4.54 ± 8.74%) (mean ± SEM, *n* = 8, unpaired *t* test, *p < 0.05). (k) The number of transitions between two boxes of blind rats (1.50 ± 0.59) and blind rats with implants (2.38 ± 0.43) (mean ± SEM, *n* = 8, unpaired *t* test, n.s., not significant). OCT, optical coherence tomography; VEPs, visual evoked potentials.

To assess the efficacy of vision restoration in visually impaired animals using arched flexible artificial photoreceptors, the composites (3 mm × 1 mm × 60 μm) were subretinally implanted into Sprague–Dawley (SD) rats that had been rendered blind by sodium iodate (Figure 5a,b). The artificial photoreceptor is implanted to replace the dysfunctional photoreceptors of the natural retina by converting light into electrical signals to stimulate bipolar cells. Postoperatively, the artificial photoreceptor remains clearly visible within the subretinal cavity without any signs of delamination or wrinkling up to 2 weeks after the surgery (Figure 5c). Additionally, no incidence of bleeding or inflammation indicates good postoperative recovery. Long‐term stability of the artificial photoreceptor within the retina has been confirmed through optical coherence tomography (OCT) images at 2 weeks and 3 months following implantation (Figure 5d and Supporting Information S1: Figure S16), demonstrating excellent biocompatibility and biosafety of flexible arched artificial photoreceptors.

We conducted an assessment of visual evoked potentials (VEPs), which are cortical responses elicited by visual stimulation, to provide objective information on the functionality of the visual pathway (Figure 5e).^[39]^ In the experiment, the right eyes of the blind rats implanted with the flexible arched artificial photoreceptor served as the experimental group, while the left eyes with no implants constituted the control group (Figure 5f). It is evident that the VEPs of the right eyes (73.15 ± 6.00 μV) show enhancement compared with those of the left eyes (36.89 ± 1.98 μV) under light stimulation (470 nm, 8 mW cm^−2^) (Figure 5g). The recorded improvement is statistically significant, indicating that flexible arched artificial photoreceptors effectively stimulate cortical response in blind rats as subretinal implants.

Finally, the in vivo behavioral responses of blind rats implanted with artificial photoreceptors were evaluated using a light‐dark box test (Figure 5h). Leveraging the nocturnal tendency of rodents to prefer darkness and avoid bright conditions, this test was designed to assess the light sensitivity of rats.^[20]^ The tests were conducted before and after the single‐eye implantation of artificial photoreceptors in blind rats, with the pre‐implantation period serving as the control group and the post‐implantation period serving as the experimental group. Two parameters were assessed: the latency period (the time it takes to enter the dark box for the first time) and the percentage of time rats spent in the dark box. Figure 5i,j shows that blind rats with implants have a significantly shorter latency period and spend a longer percentage of time in the dark box compared with blind rats. In addition, there was no obvious difference in shuttle counts between the two groups (Figure 5k), proving that the implantation did not negatively impact the rats' motor abilities. The results indicate that blind rats with implants are able to perceive light stimulation and behave innately on account of artificial photoreceptors.

## 3 CONCLUSION

In summary, we integrate LLCP, P(VDF‐TrFE) and metal electrodes to develop flexible arched artificial photoreceptors which exhibit robust photoelectric responses under weak light. The unique arched structure facilitates the concentration of photo‐induced stress within the LLCP layer, thereby amplifying the piezoelectric response of the P(VDF‐TrFE) film and enhancing the electrical output of the composite. Upon exposure to 470 nm light at 8 mW cm^−2^, an open‐circuit voltage of 17.51 ± 0.60 V was achieved, originating from light‐stress‐electricity conversion. By integrating individual artificial photoreceptors into a pixelated matrix, advanced visual functions including detection of light direction, recognition of complex patterns with varying brightness and information decoding are able to be realized. More importantly, the flexibility, repeatability, stability, and biocompatibility of the flexible arched artificial photoreceptor make it a promising candidate for retinal implant applications aimed at vision restoration. The electrophysiological detection and animal behavior analysis prove that subretinal implantation of artificial photoreceptors successfully elicits cortical responses and restores the visual response of blind rats. We believe this work will pave the way for the development of implantable artificial photoreceptors capable of restoring vision under weak‐light conditions and highlight the great potential of LCPs in next‐generation implantable flexible electronic devices.

## 4 EXPERIMENTAL SECTION

### 4.1 Materials

P(VDF‐TrFE) (VDF to TrFE ratio: 70/30) was purchased from Kunshan Hisense Electronic Co., Ltd. LLCP was synthesized according to previously reported method.^[38]^ Rhodamine Phalloidin and Triton X‐100 were purchased from Yeasen. HUVECs were obtained from Cell Bank, Chinese Academy of Sciences. Dulbecco's modified Eagle medium (DMEM), penicillin‐streptomycin antibiotic mixture, paraformaldehyde, and phosphate‐buffered saline (PBS) were purchased from Beyotime. Chlortetracycline was purchased from Hubei Qianjiang Pharmaceutical Co., Ltd. Tropicamide eye drops were purchased from Beijing Twinluck Pharmaceutical Co., Ltd. SD rats were purchased from Shulaibao (Wuhan) Biotechnology Co., Ltd.

### 4.2 Device fabrication

LLCP film and P(VDF‐TrFE) film were prepared by previously reported method.^[31]^ Metallic silver was deposited as the top and bottom electrodes on both sides of the P(VDF‐TrFE) flexible film via magnetron sputtering with a photomask. The magnetron sputtering was conducted in a direct current mode at room temperature for 300 s with the gas flow rate at 50 mL min^−1^ and power at 55 W. Subsequently, the top and bottom electrodes were connected to the positive and negative poles of the high‐voltage source by conductive silver tape, respectively. The film was immersed in silicone oil and polarized with a direct current electric field at 75 MV m^−1^ for 5 min by a withstand voltage tester (Nanjing Changsheng Instrument Co., Ltd., LTDCS2671BX). After polarization, the electrodes were short‐circuited to remove residual charges on the surface. The LLCP film was then attached to the P(VDF‐TrFE) film and heated at 95°C for 3 min to ensure close adhesion. Finally, the composite was pressed on an arched mold and applied with a pressure of 150 N cm^−2^ at 60°C for 5 min to obtain the flexible arched artificial photoreceptor. The fabrication of the pixelated matrix was similar to the single artificial photoreceptor, with the distinction lying in the employment of different electrode photomasks.

### 4.3 Fabrication of photomasks and arched molds

The electrode photomask was designed by Auto CAD and processed from an aluminum plate with a thickness of 0.3 mm by a computer numerical control engraving machine (CNC, Guangzhou Yuding Technology Co., Ltd., YD4030). The fabrication of the patterned photomask was the same as the above. The male mold of the arched matrix was polytetrafluoroethylene processed by CNC, while the female mold was prepared using the one‐time replication method with a high‐temperature‐resistant epoxy resin.

### 4.4 Characterization of materials

The cross‐sectional morphology of the artificial photoreceptor was observed by scanning electron microscopy (Zeiss, GeminiSEM 300). A 470 nm visible light lamp (CCS, PJ‐1505‐2CA, HLV‐24BL‐3W) was used as the blue light source. The infrared thermography was measured by infrared thermal imager (Teledyne FLIR, E40). The short‐circuit current and open‐circuit voltage of the artificial photoreceptors were tested using a digital source meter (Keithley, 2602B). The position of the artificial photoreceptor implanted in the retina of rats was observed by OCT (Zeiss, PRIMUS 200).

### 4.5 Finite element analysis of stress distribution

The simulation of the stress distribution in the flexible arched artificial photoreceptor was conducted using the finite element analysis software Abaqus. The model dimension was 5 × 5 mm divided into 1640 hexahedral grids. The model was a two‐layer structure. The upper layer was LLCP with an elastic modulus of 300 MPa, a Poisson's ratio of 0.49 and a thickness of 20 μm. The lower layer was P(VDF‐TrFE) with an elastic modulus of 800 MPa, a Poisson's ratio of 0.49 and a thickness of 40 μm. The contact surface between two layers was hinged. The applied stress was 0.12 MPa.

### 4.6 Cultivation of cells

HUVECs were seeded in dishes on the LLCP, P(VDF‐TrFE) or glass layer and then cultured in DMEM medium supplemented with 10% fetal bovine serum and 0.1% penicillin‐streptomycin antibiotic mixture at 37°C under a fully humidified atmosphere containing 5% CO_2_.

### 4.7 Specific fluorescence detection

HUVECs grown on different layers were fixed with 4% paraformaldehyde for 15 min and then permeabilized with 0.5% Triton X‐100 for 5 min. Rhodamine phalloidin was used to stain the F‐action of cells for 30 min to visualize the cytoskeleton, and then the DAPI staining solution was used to stain the cell nucleus for 3 min. Cells were observed by a confocal laser scanning microscope (Zeiss, LSM710) after adding 1 mL PBS.

### 4.8 Implantation surgery procedures

The SD rats were injected with 40 mg kg^−1^ sodium iodate through the tail vein to build a blind rat model. Before implantation, ketamine hydrochloride (80 mg kg^−1^) and xylene (12 mg kg^−1^) were injected into SD rats for anesthesia. The conjunctiva of the scleral limbus of the rat was cut to expose the sclera. A small incision of about 1 mm was cut 2 mm behind the scleral limbus, and 1–2 μL of saline solution was injected through a microinjector. Then, the artificial photoreceptor (1 × 3 mm) was implanted with the metal electrode facing the natural retina. The wound was closed by thermocoagulation. The whole experimental process was performed in a sterile environment with the help of an ophthalmic surgical microscope, and eyes of SD rats were kept wet. After the surgery, chlortetracycline ointment was used to prevent eye infection. The implantation position was evaluated by OCT. Before taking fundus photos, the rats were anesthetized, and 1% tropicamide eye drops were dripped into their eyes to dilate the pupils.

### 4.9 VEPs detection

SD rats were first anesthetized and fixed to install test electrodes. The recording, ground and reference electrodes were inserted into the occipital tuberosity, middle of the forehead and tail of SD rats, respectively, as shown in Figure 5f. The non‐test eye of the rat was covered with an opaque black cloth. The test eye was under illumination of 470 nm light (8 mW cm^−2^) while VEPs were recorded by visual electrophysiological diagnostic system (Roland Consul, RETI port/scan 21).

### 4.10 Light‐dark box test

SD rats were first placed in the dark for 300 s to adapt to the environment. The 470 nm light (8 mW cm^−2^) was turned on to illuminate the light box while the behaviors of rats within 300 s were recorded.

### 4.11 Statistics

Data are represented as mean ± SEM, with *n* as the number of independent animals. Statistical analysis was carried out using Origin software.

## CONFLICT OF INTEREST STATEMENT

The authors declare no conflicts of interest.

## ETHICS STATEMENT

All the animal protocols were approved by the Animal Care and Use Committee of Nantong University and the Jiangsu Province Animal Care Ethics Committee (Approval ID: SYXK (SU) 2017‐0046), and the methods were carried out in accordance with the approved guidelines.

## Supporting information

Supporting Information S1

Video S1

## Data Availability

The data that support the findings of this study are available from the corresponding author upon reasonable request.

## References

[1] S. Zhu , T. Xie , Z. Lv , Y. Leng , Y. Zhang , R. Xu , J. Qin , Y. Zhou , V. A. L. Roy , S. Han , Adv. Mater. 2024, 36, 2301986. 10.1002/adma.202301986 37435995

[2] J. Tang , N. Qin , Y. Chong , Y. Diao , Yiliguma , Z. Wang , T. Xue , M. Jiang , J. Zhang , G. Zheng , Nat. Commun. 2018, 9, 786. 29511183 10.1038/s41467-018-03212-0 PMC5840349

[3] A. Santos , M. S. Humayun , E. de Juan , R. J. Greenburg , M. J. Marsh , I. B. Klock , A. H. Milam , Arch. Ophthalmol. 1997, 115, 511. 9109761 10.1001/archopht.1997.01100150513011

[4] M. S. Humayun , J. D. Dorn , L. da Cruz , G. Dagnelie , J. A. Sahel , P. E. Stanga , A. V. Cideciyan , J. L. Duncan , D. Eliott , E. Filley , A. C. Ho , A. Santos , A. B. Safran , A. Arditi , L. V. Del Priore , R. J. Greenberg , A. I. S. Grp , Ophthalmology 2012, 119, 779. 22244176 10.1016/j.ophtha.2011.09.028 PMC3319859

[5] M. S. Humayun , J. D. Weiland , G. Y. Fujii , R. Greenberg , R. Williamson , J. Little , B. Mech , V. Cimmarusti , G. Van Boemel , G. Dagnelie , E. de Juan , Vision Res. 2003, 43, 2573. 13129543 10.1016/s0042-6989(03)00457-7

[6] M. M. K. Muqit , M. Velikay‐Parel , M. Weber , G. Dupeyron , D. Audemard , B. Corcostegui , J. Sahel , Y. Le Mer , Ophthalmology 2019, 126, 637. 30591229 10.1016/j.ophtha.2018.11.010

[7] K. Stingl , R. Schippert , K. U. Bartz‐Schmidt , D. Besch , C. L. Cottriall , T. L. Edwards , F. Gekeler , U. Greppmaier , K. Kiel , A. Koitschev , L. Kühlewein , R. E. MacLaren , J. D. Ramsden , J. Roider , A. Rothermel , H. Sachs , G. S. Schröder , J. Tode , N. Troelenberg , E. Zrenner , Front. Neurosci. 2017, 11, 445. 28878616 10.3389/fnins.2017.00445 PMC5572402

[8] H. Lorach , G. Goetz , R. Smith , X. Lei , Y. Mandel , T. Kamins , K. Mathieson , P. Huie , J. Harris , A. Sher , D. Palanker , Nat. Med. 2015, 21, 476. 25915832 10.1038/nm.3851 PMC4601644

[9] Z. Wang , J. Ma , J. Liu , X. Liu , Y. Zhu , H. Guan , C. Sun , B. Chu , Nano Energy 2024, 129, 110002.

[10] Y. Zhu , X. Liu , J. Ma , Z. Wang , H. Jiang , C. Sun , D. Y. Jeong , H. Guan , B. Chu , ACS Appl. Mater. Interfaces 2024, 16, 48395. 39223074 10.1021/acsami.4c12460

[11] Y. Jiang , B. Tian , Nat. Rev. Mater. 2018, 3, 473. 31656635 10.1038/s41578-018-0062-3 PMC6815105

[12] X. Zhu , F. Wang , Q. Zhao , X. Du , Adv. Funct. Mater. 2024, 34, 2314575.

[13] L. Bareket , N. Waiskopf , D. Rand , G. Lubin , M. David‐Pur , J. Ben‐Dov , S. Roy , C. Eleftheriou , E. Sernagor , O. Cheshnovsky , U. Banin , Y. Hanein , Nano Lett. 2014, 14, 6685. 25350365 10.1021/nl5034304 PMC4367200

[14] M. Tang , X. Zhang , A. Yang , Y. Liu , K. Xie , Y. Zhou , C. Wang , J. Liu , P. Shi , X. Lin , Small 2022, 18, 2105388. 10.1002/smll.202105388 34894073

[15] N. Li , Q. Wang , C. He , J. Li , X. Li , C. Shen , B. Huang , J. Tang , H. Yu , S. Wang , L. Du , W. Yang , R. Yang , D. Shi , G. Zhang , ACS Nano 2023, 17, 991.

[16] S. Francia , S. Di Marco , M. L. DiFrancesco , D. V. Ferrari , D. Shmal , A. Cavalli , G. Pertile , M. Attanasio , J. F. Maya‐Vetencourt , G. Manfredi , G. Lanzani , F. Benfenati , E. Colombo , Adv. Mater. Technol. 2023, 8, 2201467.

[17] L. Ferlauto , M. J. I. A. Leccardi , N. A. L. Chenais , S. C. A. Gilliéron , P. Vagni , M. Bevilacqua , T. J. Wolfensberger , K. Sivula , D. Ghezzi , Nat. Commun. 2018, 9, 992. 29520006 10.1038/s41467-018-03386-7 PMC5843635

[18] D. Ghezzi , M. R. Antognazza , R. Maccarone , S. Bellani , E. Lanzarini , N. Martino , M. Mete , G. Pertile , S. Bisti , G. Lanzani , F. Benfenati , Nat. Photonics 2013, 7, 400. 27158258 10.1038/nphoton.2013.34 PMC4855023

[19] D. Ghezzi , M. R. Antognazza , M. Dal Maschio , E. Lanzarini , F. Benfenati , G. Lanzani , Nat. Commun. 2011, 2, 166. 21266966 10.1038/ncomms1164

[20] J. F. Maya‐Vetencourt , D. Ghezzi , M. R. Antognazza , E. Colombo , M. Mete , P. Feyen , A. Desii , A. Buschiazzo , M. Di Paolo , S. Di Marco , F. Ticconi , L. Emionite , D. Shmal , C. Marini , I. Donelli , G. Freddi , R. Maccarone , S. Bisti , G. Sambuceti , G. Pertile , G. Lanzani , F. Benfenati , Nat. Mater. 2017, 16, 681. 28250420 10.1038/nmat4874 PMC5446789

[21] J. J. Wie , D. H. Wang , V. P. Tondiglia , N. V. Tabiryan , R. O. Vergara‐Toloza , L. Tan , T. J. White , Macromol. Rapid Commun. 2014, 35, 2050. 25339451 10.1002/marc.201400455

[22] Y. Xiong , L. Zhang , P. Weis , P. Naumov , S. Wu , J. Mater. Chem. A 2018, 6, 3361.

[23] X. Chen , S. Pan , P. Feng , H. Bian , X. Han , J. Liu , X. Guo , D. Chen , H. Ge , Q. Shen , Adv. Mater. 2016, 28, 10684. 27731536 10.1002/adma.201603618

[24] X. Huang , X. Pang , L. Qin , Y. Yu , Acta Polym. Sin. 2022, 53, 1324.

[25] H. Yu , T. Ikeda , Adv. Mater. 2011, 23, 2149. 21484890 10.1002/adma.201100131

[26] Z. Liao , F. Wang , Smart Mol. 2024, 2, e20240036. 40626274 10.1002/smo.20240036 PMC12118190

[27] S. Lv , Y. Zhang , W. Wang , S. Zhang , B. Tang , Smart Mol. 2024, 2, e20240058. 40626269 10.1002/smo.20240058 PMC12118281

[28] Y. Liu , J. Ma , Y. Yang , C. Valenzuela , X. Zhang , L. Wang , W. Feng , Smart Mol. 2024, 2, e20230025. 40625524 10.1002/smo.20230025 PMC12118170

[29] Y. Pu , X. Zhang , X. Liu , X. Zhao , Z. Yang , Y. Yu , Trans. Mater. Res. 2025, 1, 100003.

[30] X. Pang , J. Lv , C. Zhu , L. Qi , Y. Yu , Adv. Mater. 2019, 31, 1904224. 10.1002/adma.201904224 31595576

[31] B. Peng , X. Chen , G. Yu , F. Xu , R. Yang , Z. Yu , J. Wei , G. Zhu , L. Qin , J. Zhang , Q. Shen , Y. Yu , Adv. Funct. Mater. 2023, 33, 2214172.

[32] S. You , H. Shi , J. Wu , L. Shan , S. Guo , S. Dong , J. Appl. Phys. 2016, 120, 234103.

[33] W. Jung , M. Lee , M. Kang , H. G. Moon , S. Yoon , S. Baek , C. Kang , Nano Energy 2015, 13, 174.

[34] X. Yuan , X. Gao , J. Yang , X. Shen , Z. Li , S. You , Z. Wang , S. Dong , Energy Environ. Sci. 2020, 13, 152.

[35] Y. K. Fuh , B. S. Wang , C. Tsai , Sci. Rep. 2017, 7, 6759. 28754916 10.1038/s41598-017-07360-z PMC5533785

[36] J. Zhang , S. Ye , H. Liu , X. Chen , X. Chen , B. Li , W. Tang , Q. Meng , P. Ding , H. Tian , X. Li , Y. Zhang , P. Xu , J. Shao , Nano Energy 2020, 77, 105300.

[37] X. Hu , S. Yu , B. Chu , Mater. Des. 2020, 192, 108700.

[38] J. Lv , Y. Liu , J. Wei , E. Chen , L. Qin , Y. Yu , Nature 2016, 537, 179. 27604946 10.1038/nature19344

[39] O. R. Marmoy , M. T. Pompe , J. Kremers , Prog. Retin. Eye Res. 2024, 101, 101272. 38761874 10.1016/j.preteyeres.2024.101272

