# Supplementary material for: A flexible arched artificial photoreceptor constructed by photodeformable liquid crystal polymers and its application in vision restoration
Source: Smart Mol. 2026 Jan 19;4(2):e70030. doi: 10.1002/smo2.70030 (PMC13317704; doi:10.1002/smo2.70030)
Supplement: Supplementary file 1 — Supporting Information S1 [file SMO2-4-e70030-s002.docx]

Supporting Information

**A Flexible Arched Artificial Photoreceptor Constructed by Photodeformable Liquid Crystal Polymers and Its Application in Vision Restoration**

Yumeng Jiang^†^, Bo Peng^†^, Jinyu Ma, Feng Pan, Jia Wei, Lang Qin, Cheng Sun and Yanlei Yu^*^


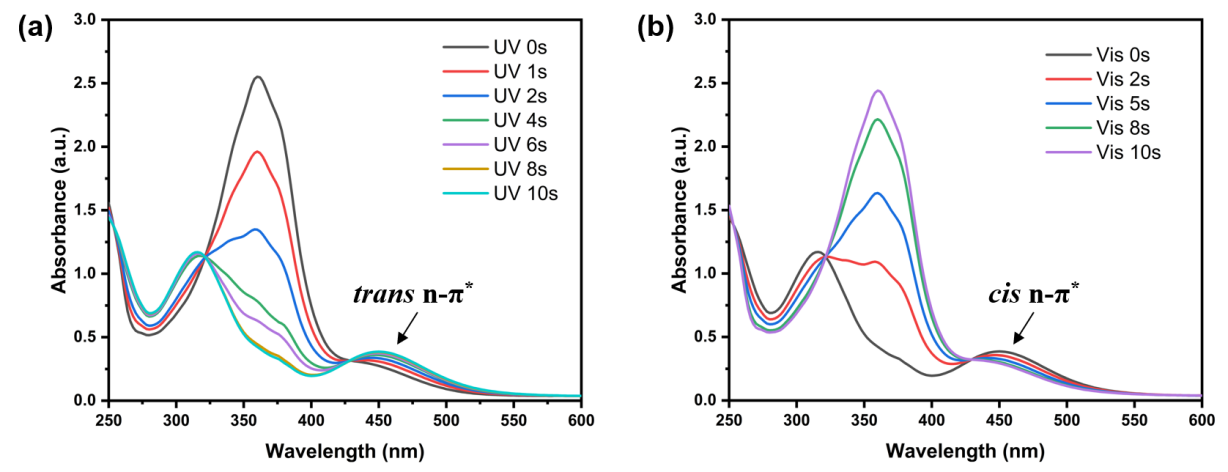


**Figure S1.** The UV-vis absorption spectra of LLCP in the dichloromethane solution upon irradiation of (a) UV light (365 nm, 80 mW cm^-2^) and (b) green light (530 nm, 80 mW cm^-2^)


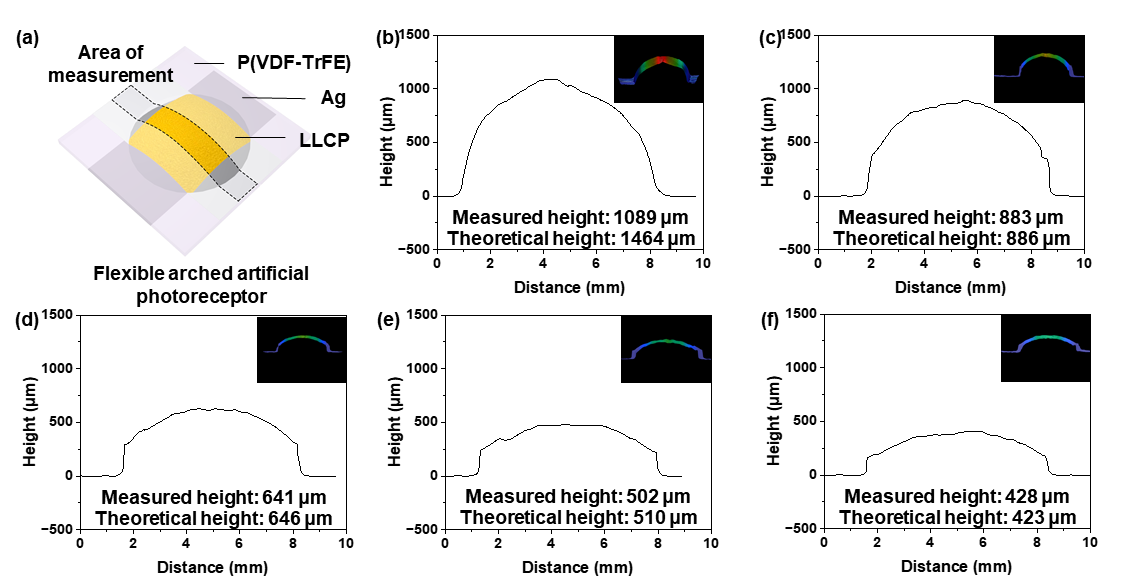


**Figure S2.** (a) Schematic illustration of the shape measurement of flexible arched artificial photoreceptors. (b-f) The shape curves of the flexible arched artificial photoreceptors whose radii of the curvature are different. Inserts indicate the photographs of the measured area. The curves indicate the central axis of flexible arched artificial photoreceptors measured after fabrication for 1 month.


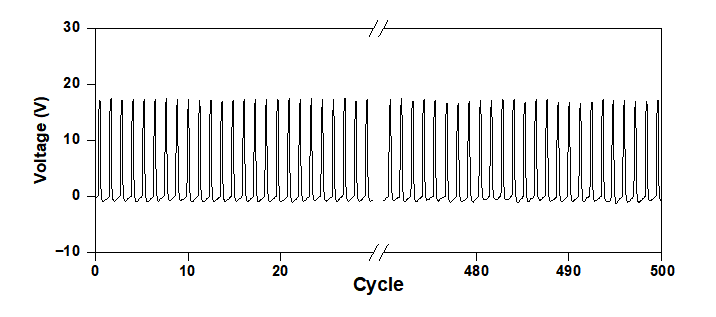


**Figure S3.** Plot showing the open-circuit voltage of the flexible arched artificial photoreceptor in 500 illumination cycles. The light intensity is 8 mW cm^-2^.


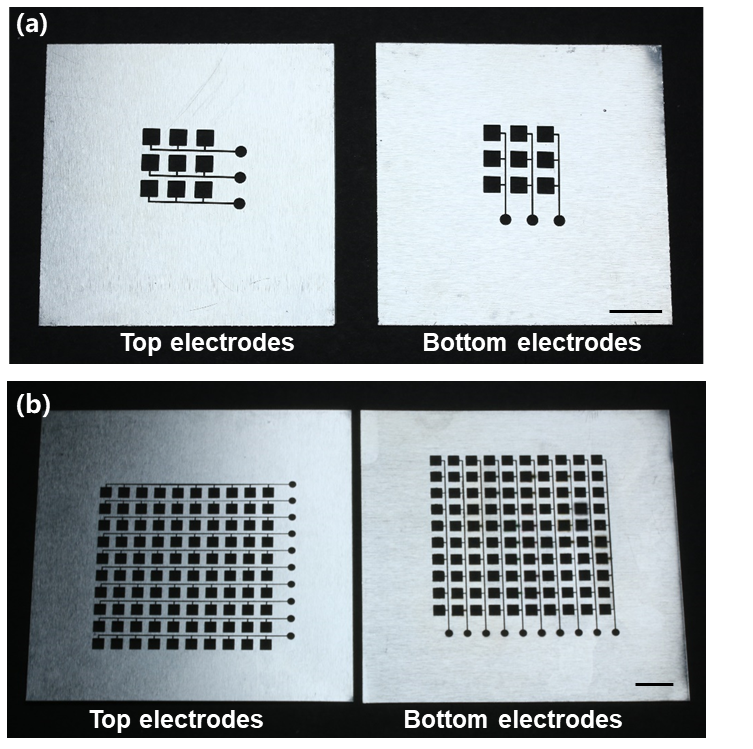


**Figure S4.** (a) Photograph of the 3 × 3 electrode photomask. (b) Photograph of the 10 × 10 electrode photomask. Scale bar: 1 cm.


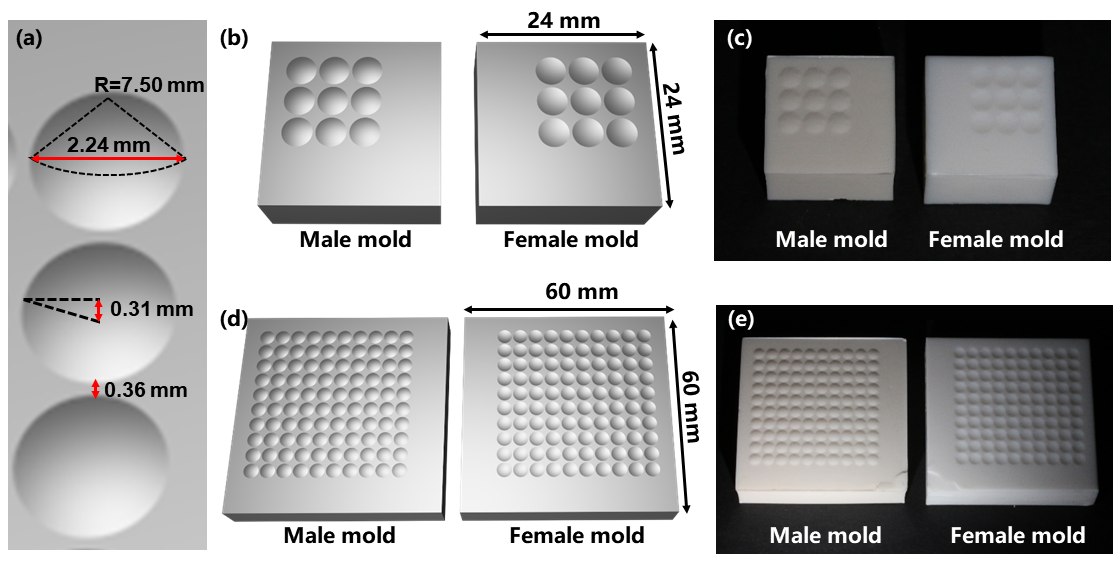


**Figure S5.** (a) Schematic illustration to show the size parameters of the arched molds. (b) The schematic illustration and (c) the photograph to show the 3 × 3 arched molds. (d) The schematic illustration and (e) the photograph to show the 10 × 10 arched molds.


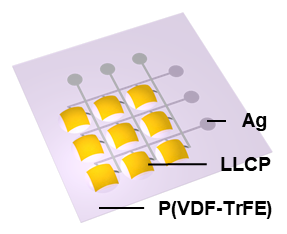


**Figure S6.** Schematic illustration to show the 3 × 3 flexible arched pixelated matrix.


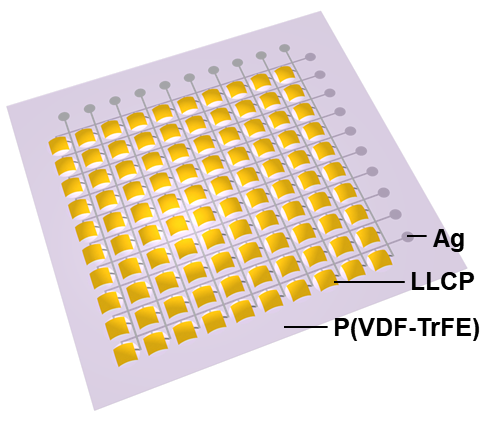


**Figure S7.** Schematic illustration to show the 10 × 10 flexible arched pixelated matrix.


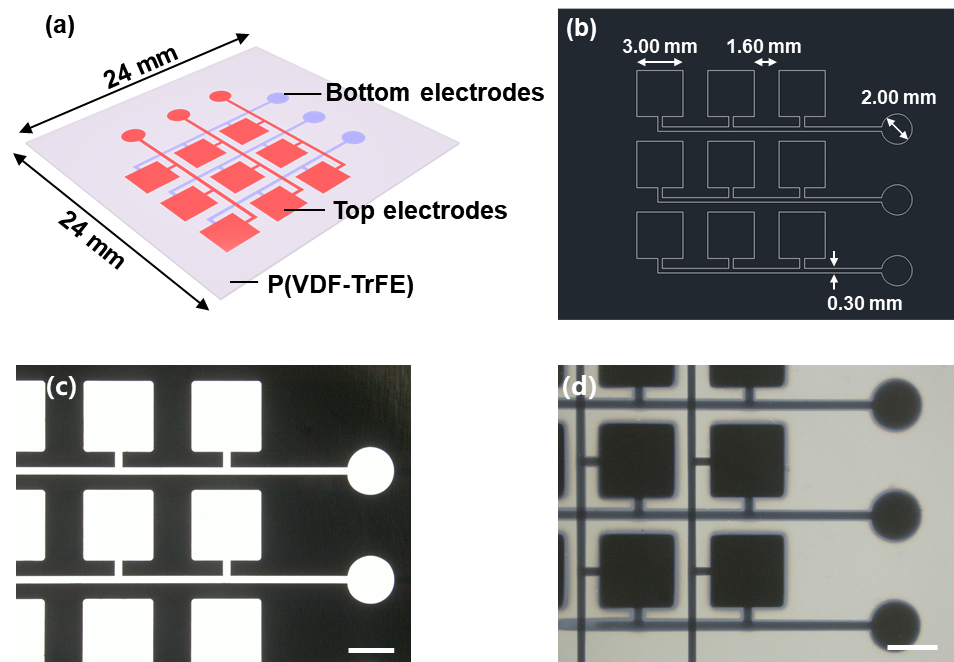


**Figure S8.** (a) Schematic illustration to show the electrodes of the 3 × 3 pixelated matrix. (b) The parameters of the electrodes and lines. (c) Photograph of the electrode photomask. (d) Photograph of the P(VDF-TrFE) film with top electrodes, bottom electrodes and lines. Scale bar: 2 mm.


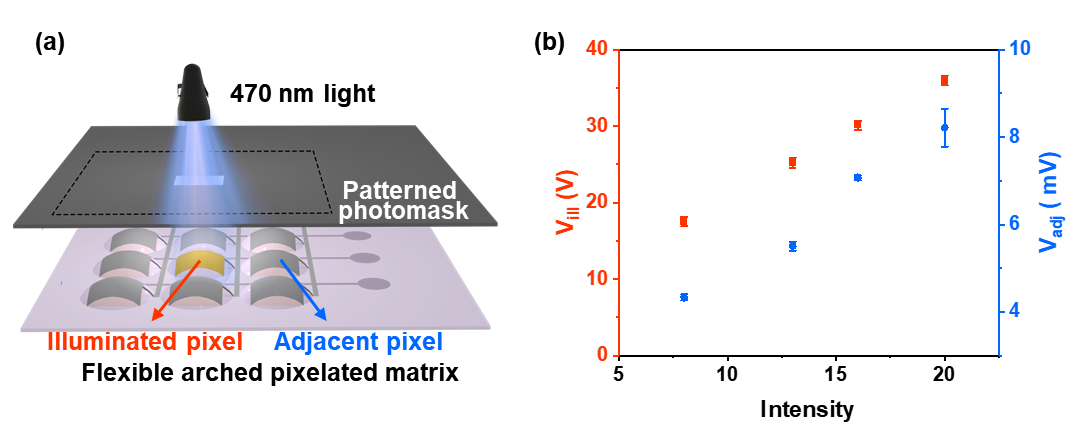


**Figure S9.** (a) Influence of the photo-induced stress generated by illuminated pixel on its adjacent pixel. (b) The open-circuit voltage of illuminated pixel (V_ill_) and adjacent pixel (V_adj_).


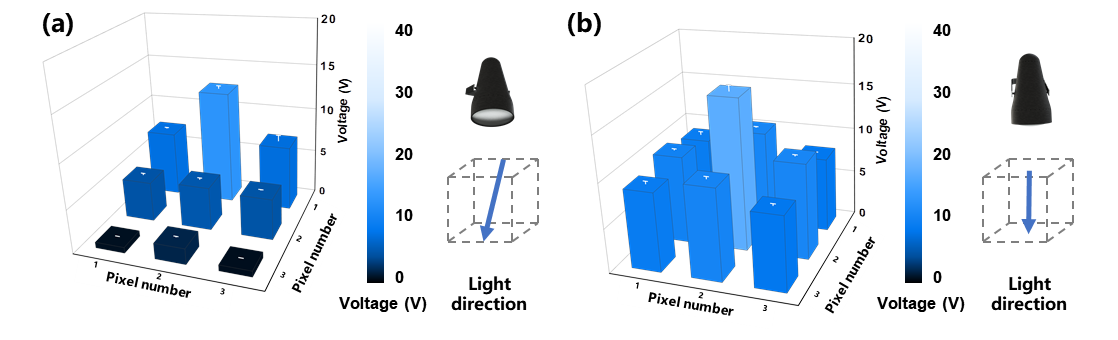


**Figure S10.** The open-circuit voltage of the 3 × 3 flexible arched pixelated matrix and light direction.


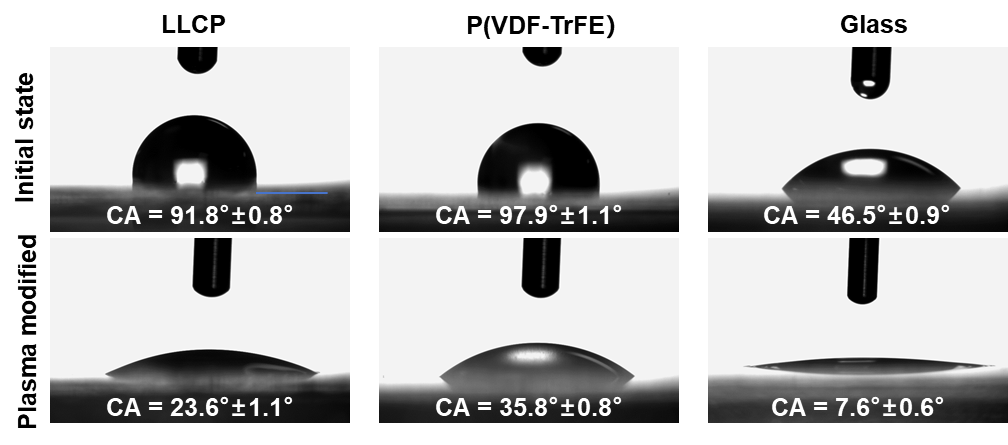


**Figure S11.** Contact angles (CAs) of a 3 μL water droplet on different materials before (top) and after (bottom) hydrophilic modification.


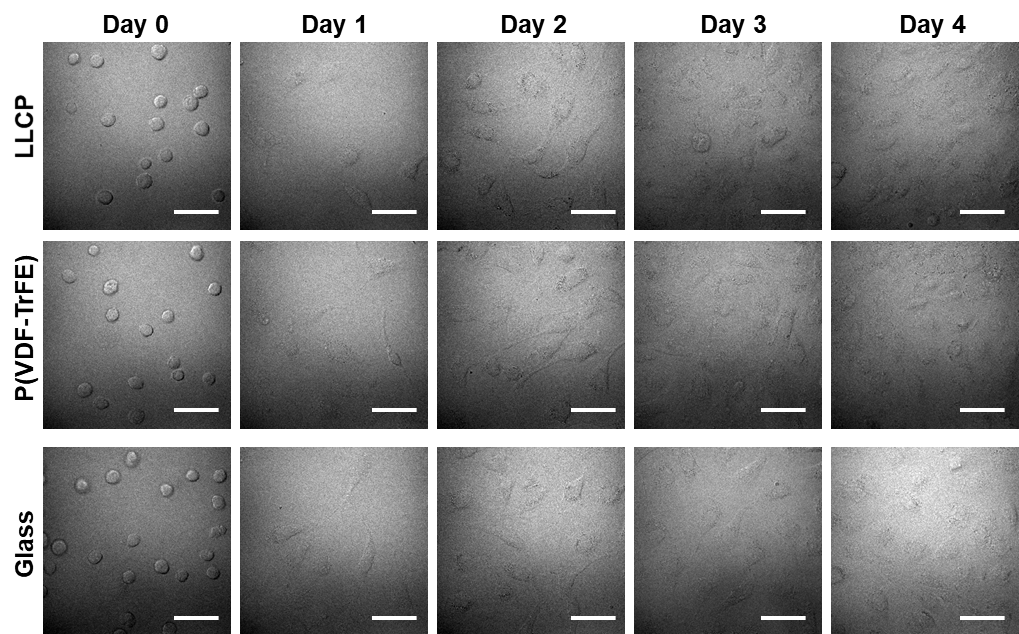


**Figure S12.** Photographs of the HUVECs on different materials. Scale bar: 50 μm.


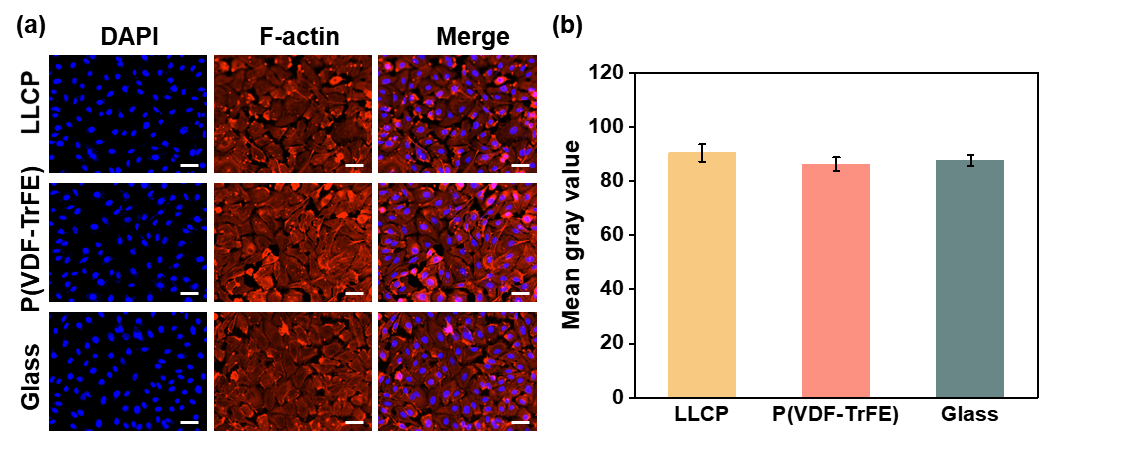


**Figure S13.** (a) F-actin specific fluorescence imaging of human umbilical vein endothelial cells (HUVECs) on the surface of different materials. Scale bar: 50 μm. (b) Mean grey value of F-actin in HUVECs on the surface of different materials.


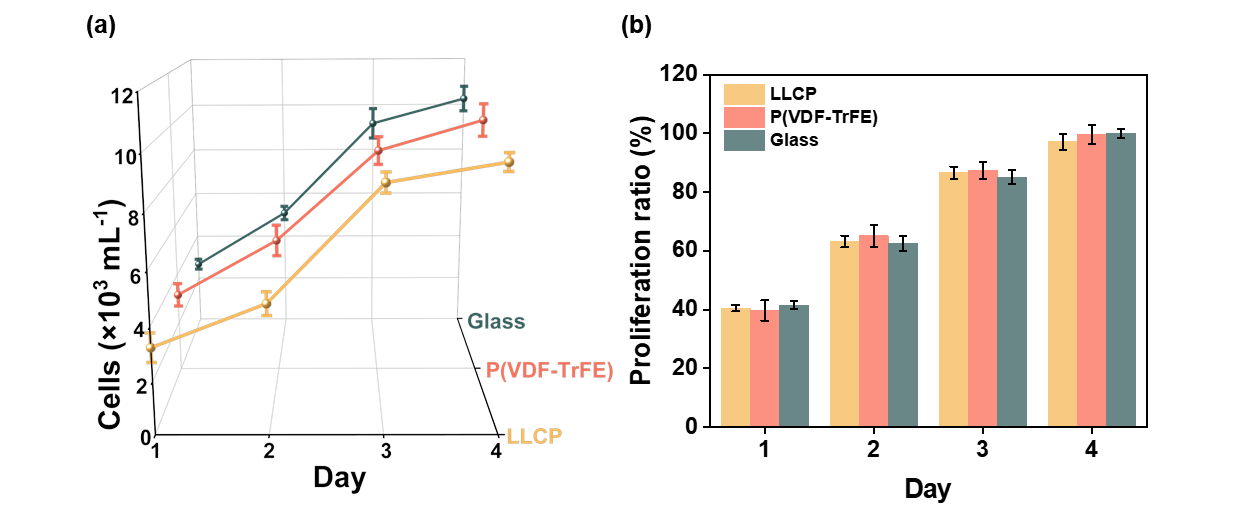


**Figure S14.** The proliferation of the HUVECs measured by (a) blood counting chamber and (b) CCK-8.


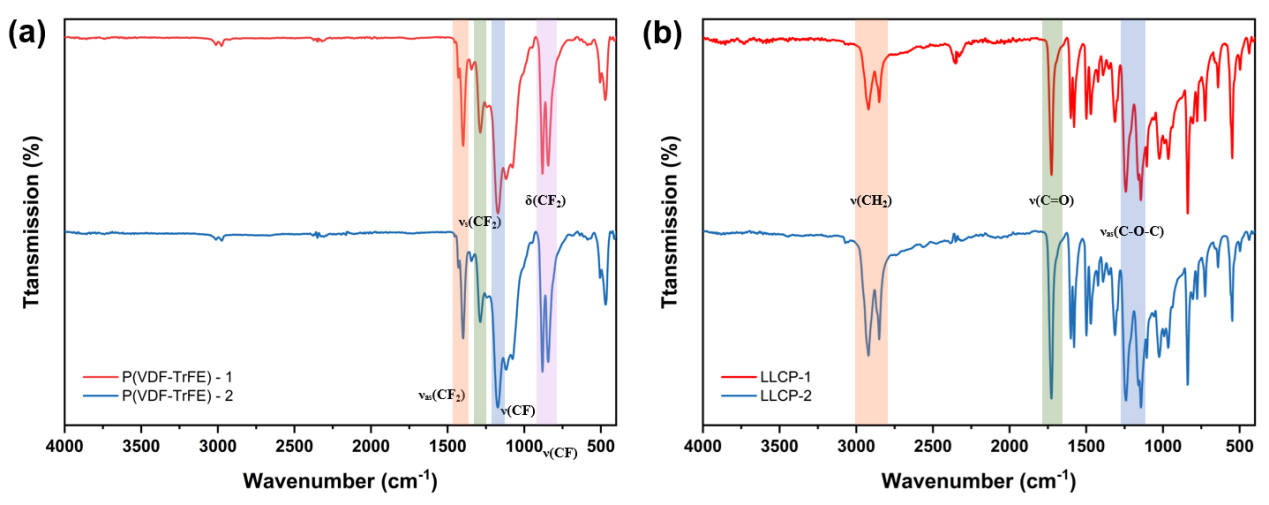


**Figure S15.** ATR-FTIR spectra of films before and after one-month immersion in physiological saline at 37 °C. (a) P(VDF-TrFE) film (before immersion: P(VDF-TrFE)-1; after immersion: P(VDF-TrFE)-2). (b) LLCP film (before immersion: LLCP-1; after immersion: LLCP-2).


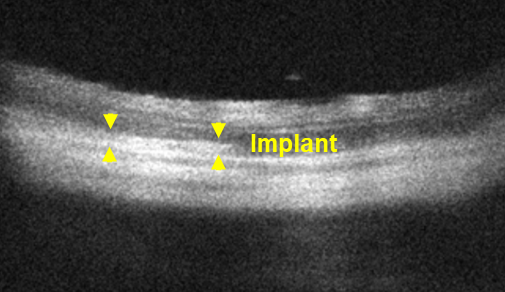


**Figure S16.** The OCT image taken 3 months after the implantation surgery of the flexible arched artificial photoreceptor.

**ASCII conversion program code:**

Dim arr() As String

Private Sub cmdClose_Click()

Unload Me

End Sub

Public Function BIN_to_DEC(ByVal Bin As String) As Long

Dim i As Long

For i = 1 To Len(Bin)

BIN_to_DEC = BIN_to_DEC * 2 + Val(Mid(Bin, i, 1))

Next i

End Function

Private Sub cmdTransfer_Click()

Dim int1 As Integer

int1 = UBound(arr)

lblTransfer.Caption = ""

If int1 <= 0 Then

MsgBox " No data can be converted", , "Information message"

Else

Dim str1 As String

str1 = ""

For i = 1 To int1

str1 = str1 & Chr(BIN_to_DEC(arr(i)))

Next

lblTransfer.Caption = str1

End If

End Sub

Private Sub Form_Load()

ReDim Preserve arr(0 To 0)

End Sub

Private Sub Form_Resize()

Image1.Left = 0

Image1.Top = 0

Image1.Width = Me.ScaleWidth

Image1.Height = Me.ScaleHeight

lblTransfer.Left = 0

lblTransfer.Width = Me.ScaleWidth

lblTransfer.Top = (Me.ScaleHeight - lblTransfer.Height) / 2

cmdTransfer.Top = Me.ScaleHeight - cmdTransfer.Height - 200

cmdTransfer.Left = Me.ScaleWidth / 2 - cmdTransfer.Width * 2

cmdClose.Top = Me.ScaleHeight - cmdClose.Height - 200

cmdClose.Left = Me.ScaleWidth / 2 + cmdClose.Width

End Sub

Private Sub ImportFile_Click()

On Error Resume Next

Dim FName As String

With CommonDialog1

.DialogTitle = "Open"

.CancelError = False

.Filter = "Text file (*.txt)|*.txt"

.ShowOpen

If Len(.FileName) = 0 Then

Exit Sub

End If

FName = .FileName

End With

Dim FileCode As Integer

Dim lineStr As String

FileCode = FreeFile

Dim intIndex As Integer

intIndex = 0

ReDim Preserve arr(0 To intIndex)

Open FName For Input As #FileCode

Do While Not EOF(FileCode)

Line Input #FileCode, lineStr

intIndex = intIndex + 1

ReDim Preserve arr(0 To intIndex)

arr(intIndex) = lineStr

Loop

Close #FileCode

End Sub

Private Sub SelectImage_Click()

On Error Resume Next

Dim FName As String

With CommonDialog1

.DialogTitle = "Open"

.CancelError = False

.Filter = " Background image Jpg (*.Jpg)|*.Jpg"

.ShowOpen

If Len(.FileName) = 0 Then

Exit Sub

End If

FName = .FileName

End With

Image1.Picture = LoadPicture(FName)

End Sub

Private Sub Form_Unload(Cancel As Integer)

MyExit = MsgBox("Confirm exit", vbExclamation + vbYesNo + vbDefaultButton, "Information message")

If MyExit = vbNo Then Cancel = True

End Sub

**Movie S1.** The light-dark box test that evaluated the in vivo behavioral responses of blind rats with and without the implantation of flexible arched artificial photoreceptors.
